# Risk Factors for Drinking Over a Woman’s Life Span

**Published:** 1994

**Authors:** Edith S. Lisansky Gomberg

**Affiliations:** Edith S. Lisansky Gomberg, Ph.D., is a professor of psychology in the Department of Psychiatry, Alcohol Research Center, University of Michigan, Ann Arbor, Michigan

## Abstract

Because many elements of a woman’s life, such as her upbringing or career, occur at different points in her life, some may be risk factors for developing problem drinking only at certain periods during the life cycle. Few long-term studies have focused on risk factors throughout the life span, leaving scientists to glean what factors they can from studies of specific age groups of women.

Clearly, not all women in a given population are equally vulnerable to becoming problem drinkers. Certain behaviors or elements of a woman’s history and genetic makeup may increase her risk of developing a harmful drinking pattern. These elements are called risk factors for problem drinking.^1^ To be most effective, efforts to prevent problem drinking should target groups of women who exhibit risk factors for developing it.

What is meant by risk factors? Ideally, with longitudinal research, which follows a group of subjects over a period of time (e.g., Fillmore et al. 1979), it would be possible to say with some certainty that “X” is an etiologic factor that frequently precedes the development of problem drinking. “X” could then be called a risk factor.

Some risk factors apparently are present throughout the female life span. Others are specific to certain periods of life, including the adolescent, young adult, middle- age, and older stages. Because longitudinal followup studies of women are scarce, researchers must cull the published literature for risk factors cited at each stage of the life span. Such factors are “markers” of increased vulnerability; that is, they often clearly are antecedent but sometimes only are correlated with the development of problem drinking and cannot definitively be called risk factors. A woman’s role in society may be a risk factor, and role is defined for both men and women primarily by age, gender, socioeconomic class, and ethnicity.

This article describes those risk factors that appear to exist for various age groups among women to increase awareness among people involved in the alcohol field about the critical role of stages in the life span. Efforts to prevent problem drinking should target women in each age group (described below) who manifest risk factors.

Why examine life stages? Issues that may be relevant to prevention and treatment of adolescents and young women may be irrelevant to prevention and treatment directed toward older women. Alcohol use and the consequences of use also appear to shift among age groups. For example, when alcoholism treatment began a half century ago, typical patients seen in treatment facilities were in their forties. For some decades now, typical patients have been younger. Of those in treatment in the United States, 35 percent are between the ages of 25 and 34, and 25 percent are between the ages of 35 and 44 (National Institute on Alcohol Abuse and Alcoholism [NIAAA] 1993). These figures, which include both men and women, demonstrate that people now begin treatment at an earlier age than they did one or two generations ago.

## Risk Factors Throughout a Woman’s Life Span

### Positive Family History

One obvious risk factor that is important in predicting a greater likelihood of problem drinking among both women and men is a family history of problem drinking (Hill 1993). Studies over time of female twins have concluded that genetics plays a major role in the etiology of problem drinking (Kendler et al. 1992).

### Peer/Spouse Pressure

In all stages throughout the life span, women consistently report the significance of the social context in which they drink. Among adolescents, data suggest that girls are more strongly influenced by peer drinking than are boys (Margulies et al. 1977) and that “group exposure” contributes significantly to alcohol misuse among female adolescents (Schulenberg et al. 1993). The drinking patterns of young adult, middle-age, and aging women also are influenced by the heavy drinking of others (Hesselbrock et al. 1984; Wilsnack et al. 1984). Heavy drinking by significant others has been reported among older female problem drinkers (Gomberg in press).

Transmission of these drinking patterns from significant others to the women must be inferred; it is reported frequently that more females than males with alcohol abuse or dependence have spouses or significant others who are problem drinkers (Gomberg 1976; Armor et al. 1976; Corrigan 1980). The important role of the husband in influencing the wife’s drinking also has been observed in countries other than the United States (e.g., Hammer and Vaglum 1989).

### Depression

At all stages over the life span, female problem drinking is linked to depression. More than 20 years ago, Schuckit (1972) made a distinction between primary and secondary alcoholism in women. Those with primary alcoholism had no history of preexisting depression; those with secondary alcoholism were depressed before a pattern of problem drinking began.

Support for a linkage between female problem drinking and depression comes from both epidemiological and clinical sources. Helzer and Pryzbeck (1988) reported that although male problem drinkers were only slightly more likely (2 percentage points) to receive a diagnosis of depression than men in the general population, female problem drinkers were more likely (12 percentage points) than were women in the general population.

Over the years, the link between female problem drinking and depression has been reported persistently in the literature (Schuckit and Morrisey 1976; Beckman 1980; Corrigan 1980; Gomberg and Lisansky 1984; Hesselbrock et al. 1985). Antecedent depression clearly is a risk factor for problem drinking. Helzer and colleagues (1991), examining Epidemiologic Catchment Area (ECA) evidence for depression co-occurring with alcoholism, found that depression precedes drinking problems in women in 66 percent of co-occurring depression and alcoholism cases examined.

Female problem drinkers who report preexisting depression differ from primary problem drinkers in several ways. They less often have a positive family history of problem drinking, have a shorter history of problem drinking, and have a more favorable prognosis (Turnbull 1988).

### Stress, Distress, and Coping

Another risk factor encountered throughout a woman’s life is her reactions to stressful events and her ability to cope with these circumstances. Stress, distress, and coping are pivotal in women’s development of problem drinking. Stress involves life events that impact a person negatively; distress is a negative response, usually in response to stress; and coping styles are used to reduce stress.

Some researchers posit that stressful events, such as trauma, are antecedent to women’s problem drinking, and some psychological theorists hypothesize that coping strategies used to modify the effects of stress may include drinking as well as seeking help and blaming others (Conte et al. 1991). Certain coping mechanisms, such as wishful thinking, escape-avoidance, and denial, may appear more frequently among female problem drinkers.^2^ The effects of dysfunctional early family life, a frequently reported source of stress, increase the likelihood not only of alcohol and other drug abuse but also of different forms of psychopathology (although it should be noted that a sizable number of women from dysfunctional or alcoholic families do not become problem drinkers) (Hill et al. 1992).

The distinction between the effects of stress, which is a life event, and distress, which is an affective response, appears in a study comparing female problem drinkers in treatment and their age peers without drinking problems (Gomberg 1989*a*). The two groups did not differ in their reports of painful early life events (e.g., they reported deaths or poverty with equal frequency), but they differed significantly in how they responded to questions about their early affective responses (e.g., “Was life unhappy or happy?”; “Did you feel unloved?”). The problem drinkers responded negatively to their traumas.

Early reports in the literature emphasized specific traumatic events as precipitating abusive drinking. In clinical treatment settings, alcoholic women were more likely to cite the influence of traumatic events (e.g., death in the family, divorce, miscarriage, or hysterectomy) than were alcoholic men. However, the interpretation of such reports, which label the traumatic event as the precipitant of drinking, has been criticized on two grounds: the terms are vague, and the reported link between unhappy life events and heavy drinking might have come from studies of a subgroup of sociopathic women for whom sociopathy produces both heavy drinking and negative events (Allan and Cooke 1985).

Another stressful life event that has been connected with women’s problem drinking is childhood sexual or physical abuse. Although presenting a history of abuse as the sole or primary etiology of female problem drinking is difficult, evidence is accumulating that indicates its significance (Coyne and Downey 1991). Childhood physical and sexual abuse, for example, has been linked with alcohol and other drug abuse (Wilsnack 1984). Once again, however, these are associations and do not explain the sequence of events leading from abuse to etiology.

One line of inquiry for researchers in this field that may further define the relationship between early life experience and problem drinking could be directed toward parental neglect and inadequate supervision in alcoholic families (Zucker and Gomberg 1986). A review of longitudinal studies has shown that “inadequate parenting” and “the child’s lack of contact with the parent” characterize the childhood homes of people more likely to develop alcohol abuse and dependence later in life (Zucker and Gomberg 1986, p. 789).

## Risk Factors Among Adolescent Girls

Although there is a vast amount of literature on adolescents’ use and abuse of alcohol, considerably less is known about adolescent girls than about adolescent boys. However, studies comparing the two groups have been increasing (e.g., Carman et al. 1983; Windle and Barnes 1988), as have occasional reports of drinking patterns and problem drinking among female adolescents (e.g., Coombs et al. 1985).

The most recent results available from surveys of 8th, 10th, and 12th graders (Johnston et al. 1993; table 1) show the prevalence of alcohol use during the last 30 days to be similar for both genders, but the percentages for females are slightly smaller than those for males. When asked about drinking five or more drinks in a row in the last 2 weeks, the same pattern appears, but the gender gap widens (more males than females drink this amount) as the survey progresses through the high school years.

### Early Versus Late Adolescence

Early adolescence differs psychologically and socially from late adolescence. This is important to consider when reviewing the environmental forces that influence adolescent drinking. For example, in early adolescence, parental influence and parents as role models may be more important than they are in late adolescence. As a person moves through adolescence, the role and influence of peers become more significant (Kandel and Andrews 1987; table 2). Peer influence also seems to play a greater role in alcohol misuse among female than male adolescents, whereas male drinking appears to depend on individual susceptibility or desire to drink (McLaughlin et al. 1985). A study by Schulenberg and colleagues (1993) finds

strong evidence for the primacy of susceptibility over exposure [i.e., peer influence] in causing alcohol misuse among young adolescents. This appears to be more true for boys than for girls. . .young adolescent girls misuse alcohol because of both individual susceptibility and group exposure (p. 8).

### Risk Factors

Many risk factors emerge from the literature for adolescent problem drinking, but no particular one appears to be typical of this problem. Instead, it appears that the more risk factors manifested by the adolescent, the more likely he or she is to engage in problem drinking (table 2).

#### Behavior Problems

One risk factor that appears most relevant for adolescent girls is behavior problems, variously described as antisocial or aggressive behavior (Zucker and Fitzgerald 1991) or as deviance. These behaviors include shoplifting; vandalism (Coombs et al. 1985); temper tantrums (Gomberg 1988); behavioral problems in early childhood; and a variety of other antisocial, acting-out behaviors. Also, rejection of authority and heightened impulsivity and aggressiveness are part of the behavioral patterns that have long been recognized as risk factors for or predictors of heavy alcohol use (Braucht et al. 1973; Windle 1990).

#### School Problems

Frequent school absences and low educational aspirations have been cited as possible clues to problem drinking among adolescent girls (Newcomb et al. 1987). Poor school performance, truancy, and dropping out are examples of such risk behaviors (Ellickson and Hays 1991; Zucker and Fitzgerald 1991). A study of alcoholic women in their twenties in treatment showed that 26 percent had dropped out of school compared with 15 percent of a matched control group (Gomberg 1986). Significantly more young alcoholic women had had trouble with school authorities, suspensions, and expulsions than the control group.

#### Family History and Environment

A family history positive for alcohol abuse and dependence and a dysfunctional early family environment surface as factors involved specifically in the development of problem drinking among adolescent girls. A dysfunctional family environment is characterized by “heightened marital conflict” between parents and “inadequate parenting” (Zucker and Fitzgerald 1991). Consistently, adolescents who abuse alcohol report very limited attachments to their parents. Young female problem drinkers frequently describe unhappy childhoods, insufficient parental attention, feelings of being unloved or unwanted, and lack of closeness to their mothers (Gomberg 1986). However, a dysfunctional early family environment does not inevitably produce psychopathology; it only increases the likelihood. Some of the nonalcoholic young women in one study (Gomberg 1986) reported a positive family history for alcohol or other drug problems or both.

#### Early Alcohol Intoxication and Marijuana Use

Newcomb and colleagues (1987) have connected experiencing alcohol intoxication early in life with problem drinking among young women. Such early intoxication also was the strongest predictor of early onset of problem drinking in a sample of women in alcoholism treatment (Gomberg 1988). In addition, Ellickson and Hays (1991) have shown that “offers of marijuana” ranks third as a predictor among young adolescents of later frequent or heavy use of alcohol. Early use of marijuana was a strong predictor of early onset of problem drinking in a sample of women in alcoholism treatment (Gomberg 1988). Both early alcohol intoxication experience and early use of marijuana suggest linkage with a diagnosis of antisocial personality (a disorder described in the *Diagnostic and Statistical Manual of Mental Disorders, Fourth Edition* [American Psychiatric Association 1994]), and this diagnosis and problem drinking among women often co-occur (Zucker and Gomberg in press).

#### Alcohol Expectancies

Alcohol expectancies (i.e., thoughts of alcohol as providing a high or removing inhibitions) are strong predictors of adolescent drinking behavior (Christiansen et al. 1989; Ellickson and Hays 1991; Reese et al. 1994). Christiansen and colleagues (1989) described expectancies about alcohol as “a theoretically active (causal) variable” (p. 98) (or one that has a direct cause-and-effect relationship) in predicting alcohol consumption and behavior problems.

#### Other Factors

Many authors have described the general influences of inadequate coping devices, alienation, and “basic neurotic tendencies” as risk factors applying especially to adolescent girls (Braucht et al. 1973). Windle and Barnes (1988) have reported that girls show a more direct relationship between distress symptoms and drinking than do boys.

## Risk Factors Among Young Adult Women

The twenties and thirties are years in which shifts in female drinking patterns occur frequently and readily (Klee and Ames 1987). These are the decades during which college attendance, career, marriage, and childbearing are likely to occur. Shifts in role and alcohol-related behaviors also are likely to occur. Some of these shifts help determine whether the woman will begin problem drinking.

### Role-Related Issues

One role influencing a woman’s drinking patterns is her position in school or her career. Women in college drink more often and in larger quantities than they will after they graduate and marry and/or find a job. Contradictory reports exist about whether married women in the workplace drink more or less than housewives (Johnson 1982). Generally it is agreed that high risk for problem drinking for women in the workplace is associated with nontraditional occupations, low-status jobs, part-time employment, and recent layoff and unemployment (Wilsnack and Wilsnack 1991).

Marital status also plays a large role in drinking patterns for adult women. Young women who are single, divorced, or separated from their spouses (i.e., those who are dating) are more likely to drink frequently and in larger quantities than married women. This also is true of women in cohabiting relationships; a report of an epidemiologic study of women’s drinking showed that the cohabiting group had more drinking problems and signs of alcohol dependence than any other marital status group (Wilsnack et al. 1984).

Both clinical and epidemiologic data have consistently revealed strong relationships between women’s drinking and their partners’ drinking (Wilsnack and Wilsnack 1991). A husband who drinks heavily may increase the likelihood of his wife drinking heavily, but it is by no means a certainty. Despite heavy drinking by their husbands, a group of blue-collar wives (average age 37.5 years) surveyed, currently drank moderately or not at all (Klee and Ames 1987). The authors explained the patterns by suggesting that women’s mothers served as role models or that the women accepted all-male social drinking or both. Whether this is characteristic of social life and marital relationships among blue-collar workers and whether it differs among white-collar families remains to be seen.

### Health-Related Issues

It is generally believed that women who are experiencing reproductive disorders, and therefore cannot become mothers, may be at risk for heavy or frequent drinking. In this case, however, it is difficult to pinpoint which comes first; that is, heavy drinking may contribute to reproductive problems, or the reproductive dysfunction itself may bring on heavy drinking. Women with reproductive dysfunction deal with such problems in a variety of ways, and the ones who cope without using alcohol do not appear in clinical data. Nevertheless, a significant relationship has been shown between miscarriages and hysterectomies on the one hand and heavy drinking among alcoholic women in treatment on the other (Gomberg 1986).

Between the ages of 15 and 75, women see their physicians more frequently than do men, with the peak difference occurring in the age group 25–44 (child-bearing years) (DeLozier and Gagnon 1991). Because women tend to use health care more (DeLozier and Gagnon 1991), health care personnel should be sensitive to messages received from women. Women typically do not, for example, report a drinking problem per se to a physician; they are more likely to report symptoms, such as gastric difficulties, insomnia, or depression. Family practice physicians, obstetricians/gynecologists, and other health care personnel should be aware of female problem-drinking indicators and be able to offer appropriate referrals when the cues are present.

### Lifestyle Patterns

Problem drinking among adult women also has been connected with their lifestyle patterns, particularly those relating to other drug use. Female problem drinkers in treatment are much more likely to be smokers than are age-matched nonalcoholic controls (82 percent versus 34 percent), and the use of both prescribed psychoactive drugs and banned substances is significantly greater among problem drinkers (Gomberg 1989*b*).

Lifestyle patterns also include public drinking. Younger women are more likely to drink in public than older women, with consequences including driving offenses, other problems with legal authorities, and vulnerability to assault (Gomberg 1990).

### Focusing on the Thirties

Although some research has been conducted on the transition from adolescence to young adulthood, the thirties as a decade in peoples’ lives is rarely investigated. Yet there are indications that for women it may be a troubling decade. Comparing men’s and women’s drinking across the adult life course, Fillmore (1987) reported that the gender ratio for risk of alcohol problems, although always greater for males, converges in the decade of the thirties. Gomberg’s study (1986) of female problem drinkers in their twenties, thirties, and forties showed intense conflict and emotional distress among women in their thirties.

It is wise to keep in mind the fluidity of this life stage. Scarf described the decade of the thirties this way (1980):

Issues of the thirties were, frequently, the mistakes that had already been made and the payment that had been exacted, an “I’ve been cheated” sense that the fantasies and dreams of girlhood had not been and might never be satisfied (p. 7).

Up to now, no one has shown how these indications of inner turmoil interact with risks of problem drinking. Developmental research about the effects of role changes and drinking patterns in the decade of the thirties is needed.

## Risk Factors Among Middle-Age Women

For women, the middle-age years, ages 40 to 59, are thought to entail “the loss of certain identity-conferring roles or ways of being” (Scarf 1980, p. 7). Although such a view is culturally determined, the fact remains that women see these years as involving loss of their youthful attractiveness and departure of their children. If, in addition, a marriage or long-term relationship breaks up during these years, the effects may indeed be painful. Wilsnack and Cheloha (1987) posited a link between loss of parenting or spousal roles and problem drinking in this age group.

In one study, 301 alcoholic women (between the ages of 20 and 50) in treatment were compared with age-matched, non-alcoholic control women (Gomberg 1986). The results showed demographic differences between the women in their forties who were problem drinkers and the matched controls. Supporting the importance of parental and spousal roles, more women who were problem drinkers than controls reported marital disruption and “empty nest” status (i.e., more problem drinkers reported that children had left home recently), but the largest difference emerged in employment status. More than 70 percent of the nonalcoholic controls were working, compared with 40 percent of the problem drinkers. It is not possible to ascribe any cause-effect to these numbers because two possible explanations exist: homemakers who are not working outside the home may be more likely to drink because of boredom, or women in their forties who are problem drinkers may be less likely to seek employment (table 3).

Several life event patterns that appear in other age groups but are apparently of greater importance in middle-age problem drinkers include the following:

- Minimal likelihood of acquiring new roles, new job, or new interests (i.e., failing to adapt to aging) (Gomberg 1986)
- “Empty nest” status, distress, and feelings of abandonment (Gomberg 1986)
- Heavy spousal drinking and marital disruption (Moos et al. 1990)
- Most frequent drinking pattern engaged in is at home and often alone (Gomberg 1993)
- Abuse of prescribed psychoactive drugs (Gomberg 1993)
- Patterns of comorbidity, including a previous or current diagnosis of depression, an eating disorder, a phobia, a panic disorder, or an anxiety state (Hesselbrock et al. 1985; Helzer and Pryzbeck 1988; Ross et al. 1988; Robins et al. 1989; Lewis and Bucholz 1992; NIAAA 1993).

## Risk Factors Among Older Women

Many cross-national epidemiologic data as well as clinical data show older people (over age 55)^3^ as more abstinent, drinking less, and manifesting fewer alcohol-related problems than younger people. Although rates of heavy and problem drinking among older people drop, alcohol-related problems continue, and older people, both men and women, do display problem drinking behavior (Welte and Miranda 1992). It has been suggested that for older women, there may be larger numbers involved in prescription drug abuse than in alcohol abuse (Glantz and Backenheimer 1988), but clearly, both types of abuse exist in this segment of the population.

Problem drinkers in the older age group can be classified as “early onset” (before age 40) and “late (or recent) onset” (after age 40) drinkers. Some evidence suggests that in this age group, women are more likely to fall into the latter category. National survey data from the ECA studies show that relatively few male problem drinkers (less than one-third) had onset after age 40. In contrast, more than one-half of the women surveyed had late onset of their problem drinking (Holzer et al. 1984).

A more recent study of older alcohol abusers (Gomberg in press; table 4) reported similar findings. Asked about age at onset, 4 percent of the older male and 38 percent of the older female alcohol abusers reported onset within the last 10 years. In another study, older women reported more recent onset than men and more use of prescribed psychoactive medication (Brennan et al. in press). They also reported more depression and, among those women with drinking problems, were less likely to seek treatment than were men.

Possible risk factors for problem drinking among older women are discussed in the following sections.

### Heavy Drinking by Significant Others

Many clinical reports document older husbands and wives, usually retired, drinking heavily and frequently together. This shared drinking has caused

unique problems in that survivors [have] not been identified as alcoholics prior to the loss of their mates. As a result their rapid decline [and the start of their heavy drinking] has often been attributed to the grief and despair of widowhood (Hubbard et al. 1979, p. 168).

Even without a history of drinking with a husband, widowhood may produce among some women an increase in alcohol intake for at least the first year after the loss. When those in the study of older alcohol abusers (Gomberg in press) who were married and had been widowed were asked about spousal drinking, only 16 percent of the male alcohol abusers compared with 36 percent of the female alcohol abusers reported their spouses as heavy drinkers.

### Problems of Retirement

This possible risk factor rarely has been studied, but some evidence suggests that retirement has a more negative impact on retired women than on retired men. Women build social networks within their places of employment, and retirement may be a stressor of significant proportions, particularly if they have limited family networks and other interests (Fox 1977). Another aspect of retirement is that for the homemaker, the husband’s retirement may be a source of stress. It will vary, of course, with the family’s finances and retirement planning, but retirement by either spouse involves important changes in family organization.

### Use of Psychoactive Drugs

Although older men and women report receiving prescriptions for psychoactive drugs to the same extent (70 percent) for both genders, women are heavier users of these drugs—as they are in all age groups (Gomberg in press). A recent Canadian survey reports that older women had the highest rate of psychoactive prescription drug use of all groups studied (Graham et al. in press), and their use was associated with widowhood, less education, poorer health, lower income, and less social support. Older women may use psychoactive drugs as their primary drug of abuse or they may be heavy alcohol consumers, using tranquilizers and sedatives in addition to alcohol.

### Moving to Retirement Communities

Many residential centers for older individuals do not facilitate drinking. However, some retirement communities, complete with golf courses and other amenities, seem to stimulate drinking and, occasionally, heavy drinking. Although this phenomenon has been described by Alexander and Duff (1988), it is not yet well explored. Because of gender differences in life expectancy, most residential arrangements for older people contain a disproportionate number of women. Thus, in studying risk factors for problem drinking among older women, the question of whether retirement communities stimulate drinking is worth examining.

## Conclusion

Two dicta emerge from a review of risk factors over the life span of women. First, although most risk factors characterize all age groups, some are unique to each stage in the life cycle. For example, inadequate control over impulses characterizes *adolescents* at risk for problem drinking, and patterns of distress are more closely tied to female than to male drinking in this age group. *Middle age* historically has been associated for women experiencing loss, depression, the “empty nest,” and menopause. Although most women in their forties and fifties handle these challenges well, for some, the events of middle age are overwhelming and distressful and may lead to problem drinking. Second, in light of the presence of more than one risk factor for each life stage, it is important to remember that the more risk factors that are present, the greater the likelihood that problem drinking will develop.

Certain questions remain to be answered about alcohol abuse and dependence among women in these age groups. For example, among adolescent girls, are those who exhibit antisocial personality behaviors and depression more likely to be abusing alcohol? Among young adult women, the question of why the gender ratio for risk of alcohol problems converges in the thirties needs exploration. Why is the thirties a decade of such great vulnerability to problem drinking for women? How do aging, depression, and alcohol intake interact among middle-age women? The question of what role distress patterns or feelings play in the etiology of problem drinking in this age group needs further study. Finally, among older women, areas that need investigation include the use of prescribed psychoactive drugs, marital patterns that lead to husbands and wives drinking together, and adaptation to widowhood.

## Figures and Tables

**Table 1:** Adolescent Alcohol Use by Grade Level

Grade	Male (%)	Female (%)
Alcohol Use in Last 30 Days		

8th	26.3	25.9
10th	41.6	38.3
12th	55.8	46.8

> 5 Drinks in a Row in Last 2 Weeks		

8th	13.9	12.8
10th	23.7	18.6
12th	35.6	20.3

SOURCE: Johnston et al. 1993.

**Table 2:** Problem Drinking Risk Factors Among Female Adolescents Ages 12 to 20

Risk Factor
Behavior problems
Problems in school
Family history positive for alcohol abuse and dependence and a dysfunctional early family environment
Early experience with alcohol intoxication
Early use of marijuana
Alcohol expectancies
Peer use of alcohol
Alienation

**Table 3:** Comparison of Female Problem Drinkers and Controls in Their Forties

Demographic Factor	Problem Drinkers (%) (*n* = 94)	Controls (%) (*n* = 50)
Marital Status		
Currently married	61	76
Divorced/separated	34	12*
Employment Status		
In the workplace	40	71
Unemployed	18	4
Homemaker	43	25*
Parental Status		
Borne children	95	88
Child left home recently	55	34*

* The difference between the findings in the two groups is statistically significant.

SOURCE: Data are from Gomberg 1990 and National Institute on Alcohol Abuse and Alcoholism 5R01 04143.

**Table 4:** Comparison of Older Male and Female Alcohol Abusers

Demographic Factor	Male (*n* = 83)	Female (*n* = 41)
Current Age (mean)	64.0 yr	66.2 yr
Mean Age at Onset of Problem Drinking	27.0 yr	46.2 yr
Current Employment Status		
Working/temporary layoff	28%	12%
Retired	63%	54%
Unemployed	10%	5%
Homemaker	0	29%
Reported Effects of Alcohol		
Felt miserable	22%	38%*
Got more depressed	37%	56%*
Got along better with people	57%	30%*
Lost my temper	30%	34%
Minor Tranquilizer Use		
Used daily, 2 weeks or more	38%	74%*
Needed larger amounts for effect	7%	37%*
Unable to keep from using	23%	53%*

* The difference between the findings in the two groups is statistically significant.

SOURCE: Data are from Gomberg in press and National Institute on Alcohol Abuse and Alcoholism 1P50 AA07378.

## Footnotes

1 This article uses the term “problem drinking” to encompass all alcohol-related disorders. The terms “alcohol abuse” and “alcohol dependence” are defined by the following criteria in the American Psychiatric Association’s *Diagnostic and Statistical Manual of Mental Disorders, Fourth Edition* (1994): heavy and frequent drinking; impaired control behavior (e.g., swearing off or trying to moderate the amount of alcohol consumed); alcohol-related consequences and continued drinking despite the consequences; and withdrawal and tolerance. Other terms such as “alcoholism” are used as they are in the studies being described.

2 Some problems exist in defining and measuring these mechanisms. For a review, see Coyne and Downey (1991).

3 Although “older people” normally would be defined as people age 60 and older, the difficulty in finding subjects for research over age 60 has caused the definition to be pushed back to age 55 for clinical research.
